# Sporadic amyotrophic lateral sclerosis (SALS) – skeletal muscle response to cerebrospinal fluid from SALS patients in a rat model

**DOI:** 10.1242/dmm.031997

**Published:** 2018-04-16

**Authors:** Shruthi Shanmukha, Gayathri Narayanappa, Atchayaram Nalini, Phalguni Anand Alladi, Trichur R. Raju

**Affiliations:** 1 Department of Neurophysiology, National Institute of Mental Health and Neurosciences, Hosur Road, Bangalore 560 029, India; 2 Department of Neuropathology, National Institute of Mental Health and Neurosciences, Hosur Road, Bangalore 560 029, India; 3 Department of Neurology, National Institute of Mental Health and Neurosciences, Hosur Road, Bangalore 560 029, India

**Keywords:** Amyotrophic lateral sclerosis, Immunohistochemistry, Electron microscopy, NMJ, Muscle atrophy, Trophic factors

## Abstract

Skeletal muscle atrophy is the most prominent feature of amyotrophic lateral sclerosis (ALS), an adult-onset neurodegenerative disease of motor neurons. However, the contribution of skeletal muscle to disease progression remains elusive. Our previous studies have shown that intrathecal injection of cerebrospinal fluid from sporadic ALS patients (ALS-CSF) induces several degenerative changes in motor neurons and glia of neonatal rats. Here, we describe various pathologic events in the rat extensor digitorum longus muscle following intrathecal injection of ALS-CSF. Adenosine triphosphatase staining and electron microscopic (EM) analysis revealed significant atrophy and grouping of type 2 fibres in ALS-CSF-injected rats. Profound neuromuscular junction (NMJ) damage, such as fragmentation accompanied by denervation, were revealed by α-bungarotoxin immunostaining. Altered expression of key NMJ proteins, rapsyn and calpain, was also observed by immunoblotting. In addition, EM analysis showed sarcolemmal folding, Z-line streaming, structural alterations of mitochondria and dilated sarcoplasmic reticulum. The expression of trophic factors was affected, with significant downregulation of vascular endothelial growth factor (VEGF), marginal reduction in insulin-like growth factor-1 (IGF-1), and upregulation of brain-derived neurotrophic factor (BDNF) and glial-derived neurotrophic factor (GDNF). However, motor neurons might be unable to harness the enhanced levels of BDNF and GDNF, owing to impaired NMJs. We propose that ALS-CSF triggers motor neuronal degeneration, resulting in pathological changes in the skeletal muscle. Muscle damage further aggravates the motor neuronal pathology, because of the interdependency between them. This sets in a vicious cycle, leading to rapid and progressive loss of motor neurons, which could explain the relentless course of ALS.

This article has an associated First Person interview with the first author of the paper.

## Abstract (precis)

**Editor's choice:** In amyotrophic lateral sclerosis, a motor neurodegenerative disease, the pathological changes of the skeletal muscle can significantly damage motor neurons, leading to progressive neurodegeneration.

## INTRODUCTION

Amyotrophic lateral sclerosis (ALS) is a motor neuron degenerative disease that affects upper and lower motor neurons, leading to perceptibly severe muscle atrophy. There is a lack of a suitable animal model for investigating the sporadic form of ALS (SALS), which accounts for 90% of cases. The complex interplay between different cell types, such as motor neurons, astrocytes, microglia, Schwann cells and skeletal muscles, in ALS pathogenesis needs to be investigated.

The loss of muscle mass is one of the hallmark features of ALS. The degeneration of motor neurons and denervation leads to atrophy of muscle fibres in ALS (Baloh et al., 2007; Brooke and Engel, 1969; Dobrowolny et al., 2008; Telerman-Toppet and Coërs, 1978; Wong and Martin, 2010). The large-calibre, fast-fatiguable motor neurons which innervate the type 2 muscle fibres are known to be selectively vulnerable in ALS (Hegedus et al., 2007; Pun et al., 2006). Following denervation, the neighbouring motor axons compensate for the loss, resulting in re-innervation of the denervated fibres by axonal sprouting, ultimately leading to grouping of muscle fibres (Baloh et al., 2007; Schaefer et al., 2005; Telerman-Toppet and Coërs, 1978). However, this compensatory mechanism fails to support regeneration, as the disease advances and results in progressive decrease in muscle strength.

Motor neurons are dependent on skeletal muscles for the continuous supply of neurotrophic factors for their survival and functioning (Henderson et al., 1998; Oppenheim, 1996). The skeletal muscle is a rich source of several neurotrophic factors. Brain-derived neurotrophic factor (BDNF) prevents motor neuron cell death (Koliatsos et al., 1993; Oppenheim et al., 1992) and mediates anti-apoptotic effects (Almeida et al., 2005). Insulin-like growth factor-1 (IGF-1) protects motor neurons during development and post-injury recovery (Neff et al., 1993), and aids in the restoration of neuromuscular junction (NMJ) function (Messi and Delbono, 2003). Glial-derived neurotrophic factor (GDNF) promotes the survival of motor neurons; overexpression of GDNF causes hyperinnervation of muscle (Henderson et al., 1994; Nguyen et al., 1998). Vascular endothelial growth factor (VEGF) has potent neurotrophic and mitogenic activity, and increases axonal outgrowth and survival of neurons (Annex et al., 1998; Sondell et al., 1999). Interestingly, deletion of the hypoxia-response element in its promoter region causes reduced VEGF expression in the spinal cord, resulting in motor neuron degeneration very similar to that seen in ALS (Oosthuyse et al., 2001). It also induces axonal regeneration after ischemic injury and muscle innervation, acting through nerve growth factor (NGF)/GDNF signalling (Shvartsman et al., 2014), and signifying the influence of VEGF on other trophic factors.

Previous studies from our laboratory on a rat model and NSC34 cell line have demonstrated the various degenerative changes upon exposure to cerebrospinal fluid (CSF) from SALS patients (ALS-CSF); these are listed in Table 1. Understanding the pathogenesis of ALS in the skeletal muscle, the end organ, appears more appropriate as it can be an efficient target for drug delivery. In this view, the current study focuses on various aspects of skeletal muscle changes in rats following intrathecal injection of ALS-CSF.

**Table 1.**
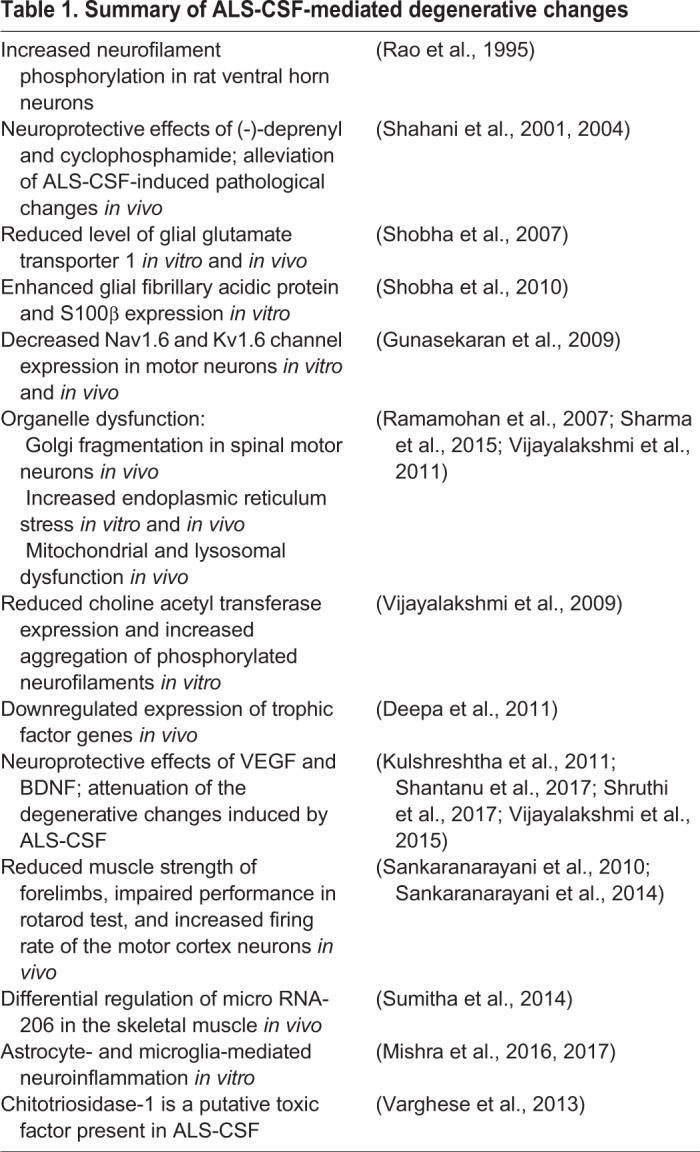
**Summary of ALS-CSF-mediated degenerative changes**

## RESULTS

### ALS-CSF induces atrophy of type 2 fibres in extensor digitorum longus muscle of rat neonates

**Fig. 1.**
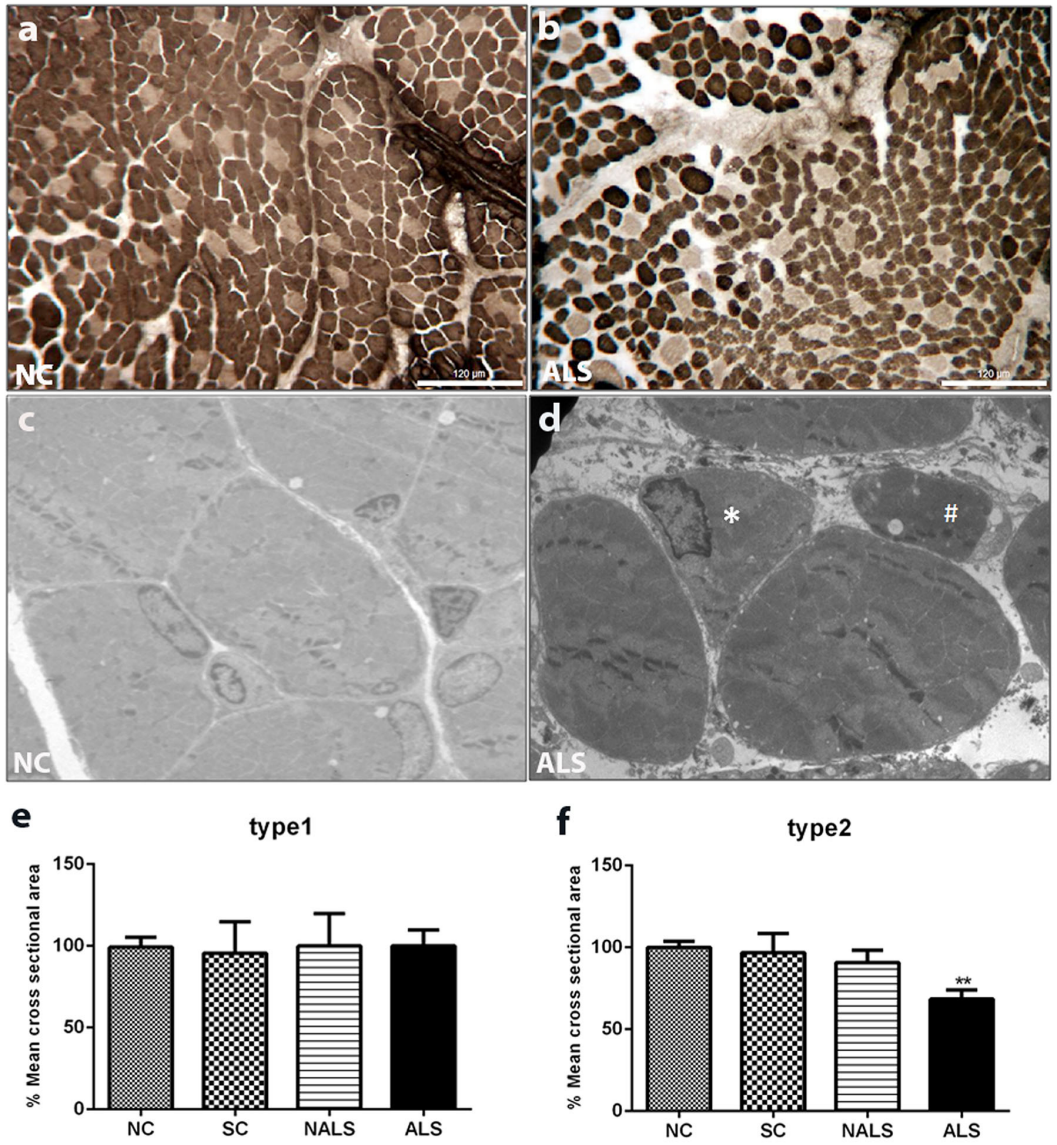
**ALS-CSF induces atrophy of type 2 fibres in EDL muscle of neonatal rats.** (A,B) ATPase-stained cross-sections of EDL muscles from NC and ALS-CSF-injected (ALS) rats. Type 1 fibres are lightly stained and type 2 fibres are darkly stained at pH 9.4. Note the altered pattern of type 1 and type 2 fibres in the ALS group compared with the NC group. Rounding, angulation and grouping of type 2 fibres are also seen. Scale bars: 120 μm. (C,D) Electron micrographs showing NC (C) and ALS (D) groups. Rounded (indicated by hash symbol) and angulated fibres (indicated by asterisk) can be seen in the ALS group, suggesting atrophy of the muscle. (E,F) Mean cross-sectional area (CSA) of muscle fibres. Note that there is no alteration in the CSA of type 1 fibres (E), whereas type 2 fibres showed a significant reduction in CSA, in the ALS group compared with the other control groups (F, **P<0.01, NC versus ALS). *n*=5 in duplicates. Statistical significance was calculated using one-way ANOVA followed by Tukey's post hoc test.

The muscle fibres are broadly divided into type 1 and type 2 fibres based on their adenosine triphosphatase (ATPase) staining. Pre-incubation with basic solution inhibits myosin ATPase in type 1 fibres, causing them to be lightly stained, whereas type 2 fibres are darkly stained at this pH, allowing their clear distinction. The normal pattern of type 1 and type 2 fibres and polygonal shape of myofibres are altered in ALS-CSF-injected animals compared with those in control groups. ALS-CSF caused significant atrophy, reflected by angulated myofibres and grouping of type 2 fibres in the extensor digitorum longus (EDL) muscle (as shown by ATPase staining at pH 9.4) in ALS-CSF-injected (ALS) rats compared with normal control (NC) rats (Fig. 1A,B). In addition, electron microscopic (EM) analysis of the muscle provides evidence of atrophy (Fig. 1C,D). Quantification of the cross-sectional area (CSA) of the muscle fibres showed a significant reduction in the size of type 2 fibres, suggesting severe atrophy, whereas type 1 fibres were unaltered (Fig. 1E,F; **P<0.01, NC versus ALS).

### ALS-CSF causes profound changes in NMJ structure in rat neonates

**Fig. 2.**
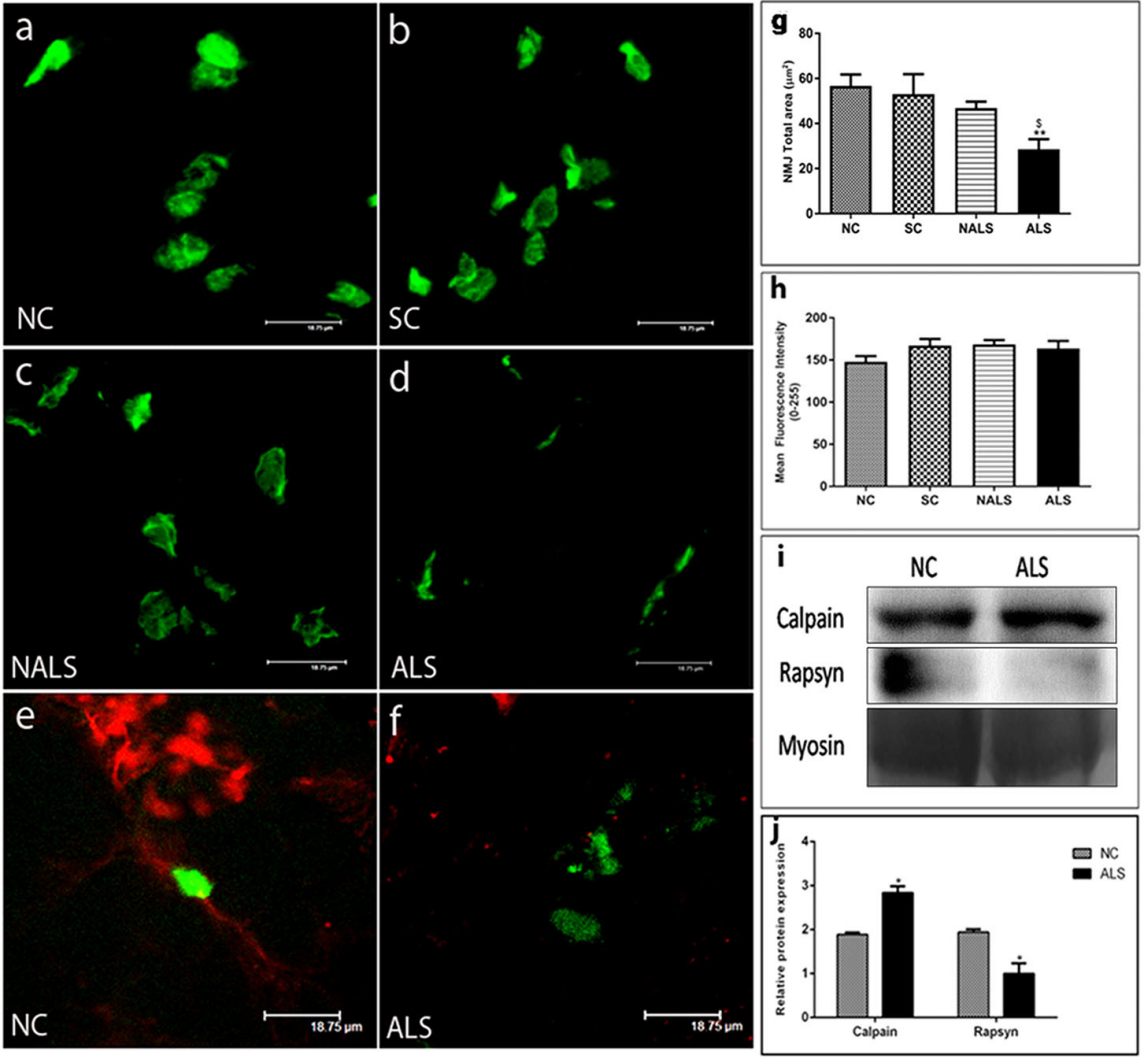
**ALS-CSF causes profound changes in NMJ structure.** (A-D) Z-stacked confocal images of Alexa Fluor 488-conjugated α-bungarotoxin-stained AChRs from NC, SC, NALS and ALS rats. Note the reduced complexity of NMJs in the ALS group compared with the control groups. Scale bars: 18.75 μm. (E,F) Nerve terminals stained with anti-200KD neurofilament (red) and postsynaptic sites with Alexa Fluor 488-conjugated α-bungarotoxin (green) to assess the status of innervation. Note the complete apposition of nerve terminals and postsynaptic site in the NC group (E), whereas the ALS group shows a lack of any juxtaposition between nerve terminals and AChR sites (F), suggesting denervation. (G,H) Quantitative analysis of total NMJ area and AChR expression in EDL muscle from NC, SC, NALS and ALS groups. The total NMJ area is significantly reduced in the ALS group compared with the control groups (G, **P<0.01, NC versus ALS; ^$^*P*<0.05 SC versus ALS), whereas the expression of AChR does not significantly differ among the groups (H). *n*=5 in duplicates. Data were analysed using one-way ANOVA followed by Tukey's post hoc test. (I) Representative western blot of rapsyn and calpain proteins normalised to myosin loading in the EDL muscle of the NC and ALS groups. (J) Quantitative representation of rapsyn and calpain expression, indicated as densitometric ratio of calpain: myosin and rapsyn: myosin. Note the significant upregulation of calpain (*P<0.05, NC versus ALS; *n*=3 in duplicates) and downregulation of rapsyn in the ALS group (*P<0.05, NC versus ALS; *n*=3 in duplicates). *n*=5 in duplicates. Data were analysed using one-way ANOVA followed by Tukey's post hoc test.

We also assessed the structural integrity of NMJs. In NC, sham control (SC) and non-ALS-CSF (NALS) rats, NMJs appear as near pretzel-shaped structures (Fig. 2A-C); in contrast, NMJs in the ALS group were found to be fragmented and diffused (Fig. 2D). There was also a reduction in the total synaptic area in the ALS group (Fig. 2G; **P<0.01, NC versus ALS; ^$^*P*<0.05, SC versus ALS). However, there was no difference in the expression of acetylcholine receptor (AChR) protein between the groups (Fig. 2H).

In addition, double labelling with anti-200KD neurofilament for nerve terminals and Alexa Fluor 488-conjugated α-bungaratoxin for NMJs showed marked denervation in the ALS group compared with the NC group (Fig. 2E,F). Further, the expression of two key proteins involved in the clustering of AChR subunits, rapsyn and calpain, was estimated. The expression of rapsyn, which helps in the clustering of AChR subunits was decreased, whereas calpain, which counteracts the rapsyn action, was upregulated, resulting in the disruption of NMJ structure (Fig. 2I,J; *P<0.05, NC versus ALS).

### Ultrastructural changes

**Fig. 3.**
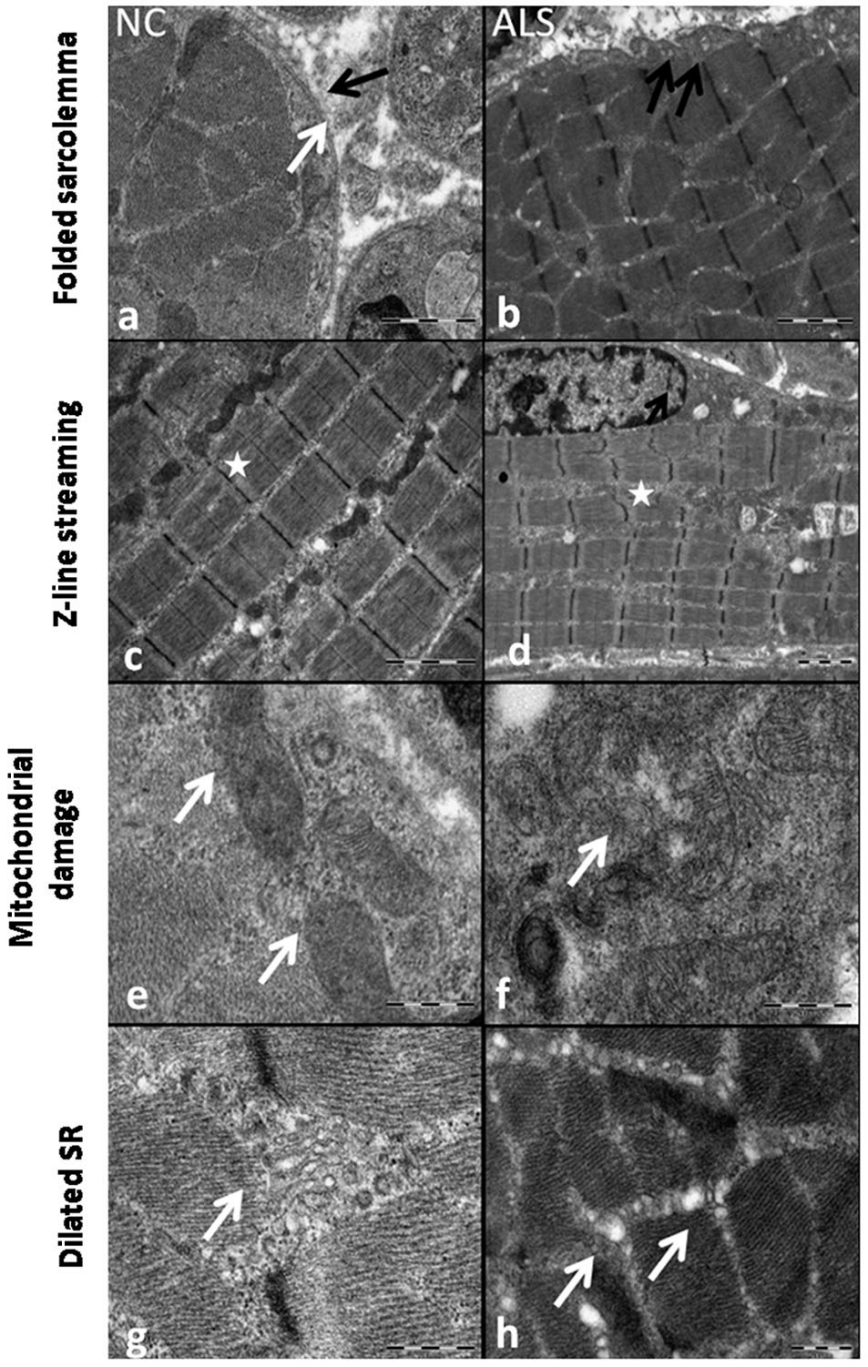
**Ultrastructural pathology.** (A-H) Representative EM image depicting the loss of sarcolemmal integrity in the ALS group. The NC group (A) shows normal sarcolemmal features with intact outer and inner layers marked by black and white arrows, respectively. However, in the ALS group, the sarcolemma was folded (B, black arrows). The longitudinal section of the skeletal muscle shows the sarcomere with perfectly aligned Z lines along with alternate dark (A band) and light (I band) bands in the NC group (C, asterisk). Note the streaming of Z-band material (D) along with misalignment of sarcomeres in the ALS group (D, asterisk). Normal mitochondria with intact membrane and cristae structure are observed in the NC group (E), whereas mitochondria have vacuolation with abnormal internal structure and ruptured membrane in the ALS group (F, white arrow). Transverse sections show reticulum structure with discretely formed cisternae in the NC group (G), compared with dilated sarcoplasmic reticulum in the ALS group (H, white arrows).

To investigate the detailed pathological changes in the skeletal muscle of the ALS animals, we carried out ultrastructural analysis of muscle tissue from NC, SC, NALS and ALS groups. The NC group showed a double-layered sarcolemma consisting of outer basement and inner plasma membranes, with a normal cytoskeletal distribution, whereas the sarcolemma was folded in the ALS group, suggesting the loss of membrane integrity and confirming atrophy (Fig. 3A,B). In addition, the normal striated appearance of the skeletal muscle was lost in the diseased group. The fibres in EDL muscles from ALS rats showed clear alterations in the banding patterns. There was a distortion of filamentous pattern with streaming of Z-band material or misalignment of sarcomeres and a focal loss of myofilaments (Fig. 3C,D). In the control groups (NC, SC and NALS), normal mitochondria with well-defined cristae and membrane structure were seen in subsarcolemmmal and intermyofibrillar regions. However, in the ALS group, the mitochondria had altered cristae, vacuolation and abnormal shape (Fig. 3E,F); additionally, many mitochondria had accumulated lipid droplets. Marked variation in the mitochondrial morphology confirmed the damage caused by ALS-CSF. In addition to mitochondrial damage, the extensive network of longitudinally oriented tubules of sarcoplasmic reticulum (SR) was damaged in the ALS group compared with the control groups. Dilated SR at the subsarcolemmmal and intermyofibrillar regions was observed, which might result in impaired calcium homeostasis (Fig. 3G,H).

### Oxidative stress

**Fig. 4.**
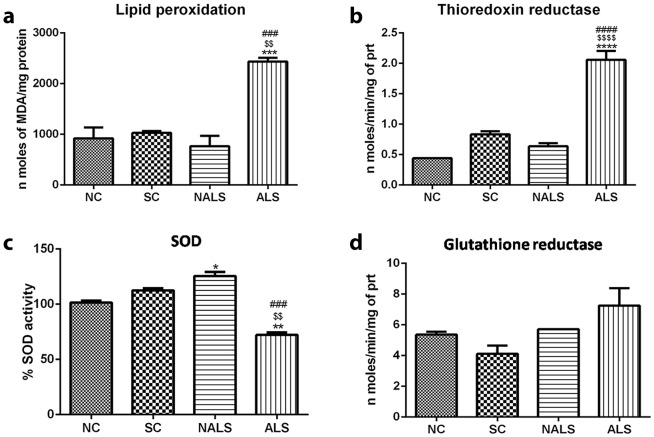
**Oxidative stress in the skeletal muscle of the ALS-CSF-injected animals.** (A-D) Assays of oxidative stress and antioxidant enzyme activity: the histograms represent lipid peroxidation (***P<0.001, NC versus ALS; ^$$^*P*<0.01, SC versus ALS; ^###^*P*<0.001, NALS versus ALS) (A), thioredoxin reductase (****P<0.0001, NC versus ALS; ^$$$$^*P*<0.0001, Sham versus ALS; ^####^*P*<0.001, NALS versus ALS) (B), SOD (*P<0.05, NC versus NALS; **P<0.01, NC versus ALS; ^$$^*P*<0.01, SC versus ALS; ^###^*P*<0.001, NALS versus ALS) (C), and glutathione reductase (D) activities (*n*=5 in duplicates). Data were analysed using one-way ANOVA followed by Tukey's post hoc test.

The ultrastructural analysis showed significant mitochondrial damage in ALS-CSF-injected rat skeletal muscle, and it could be associated with oxidative stress, which was estimated by measuring the levels of malondialdehyde (MDA), a marker of lipid peroxidation in muscle samples. The results confirmed increased oxidative stress in the skeletal muscle as we found a 2.5-fold increase in nmol of MDA/mg of protein in the ALS animals (Fig. 4A; ***P<0.001, NC versus ALS; ^$$^*P*<0.01, SC versus ALS; ^###^*P*<0.001, NALS versus ALS). We observed that muscle samples from ALS rats showed elevated MDA content, compared with those from controls. To analyse whether increased oxidative stress in ALS is linked with the antioxidant function, we assayed the activities of the constitutive antioxidant enzymes superoxide dismutase (SOD), glutathione reductase (GR) and thioredoxin reductase. There was an upregulation of thioredoxin reductase activity in the ALS group, probably as an adaptive response to increased oxidative stress (Fig. 4B; ****P<0.0001, NC, SC and NALS versus ALS). Further, SOD activity was significantly downregulated in the ALS group and, surprisingly, upregulated in the NALS group (Fig. 4C; *P<0.05, NC versus NALS; **P<0.01, NC versus ALS; ^$$^*P*<0.01, SC versus ALS; ^###^*P*<0.001, NALS versus ALS), while GR activity remained unaltered (Fig. 4D).

### Altered expression of BDNF and IGF-1 in ALS

**Fig. 5.**
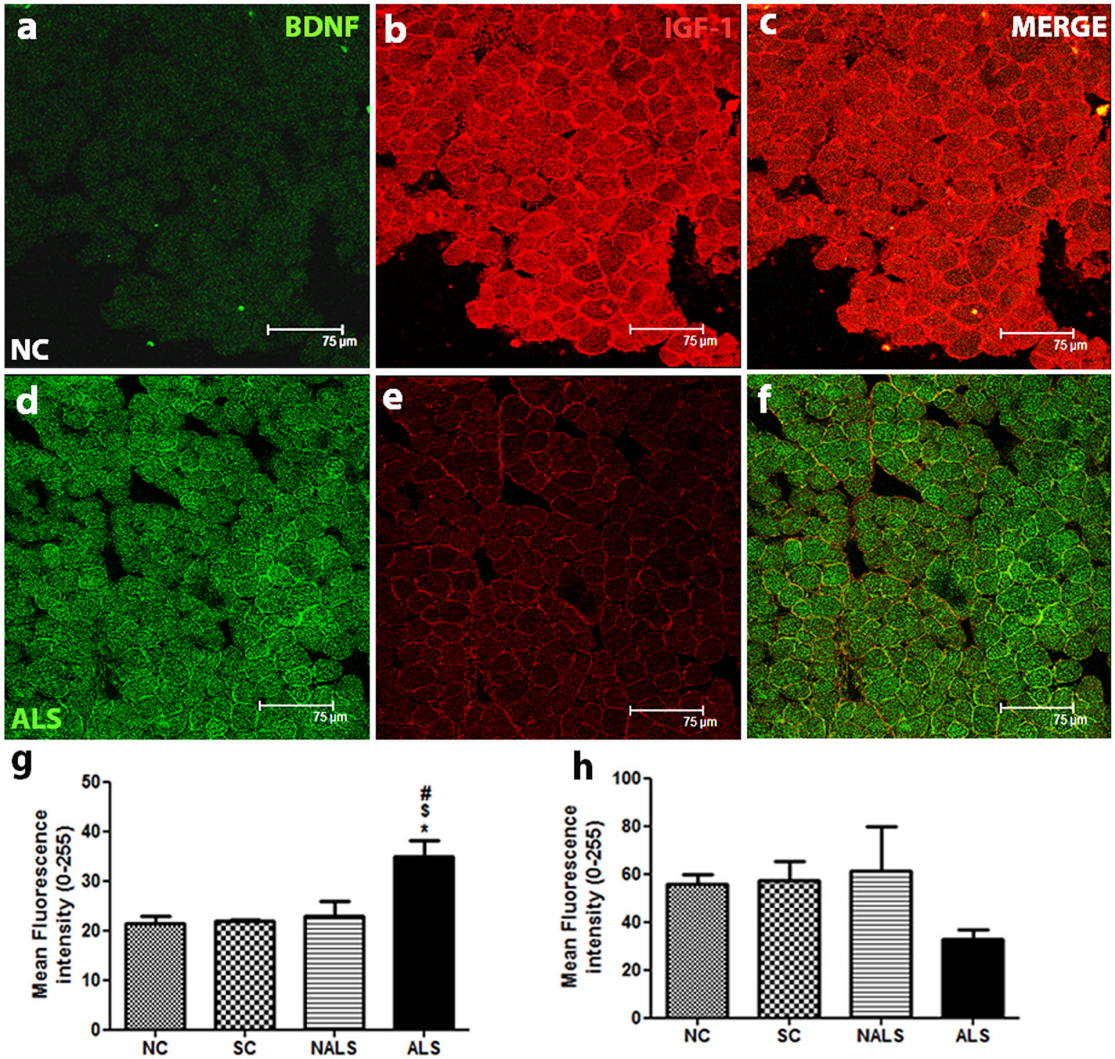
**Altered expression of BDNF and IGF-1.** (A-F) Representative photomicrographs of transverse sections of EDL muscle double labelled for BDNF (FITC) and IGF-1 (CY-3). Increased expression of BDNF and a trend towards reduction in IGF-1 levels can be observed in the ALS group. Scale bars: 75 μm. (G,H) Quantification of immunofluorescence intensity (0-255) supported the qualitative observations. BDNF was significantly upregulated in the ALS group compared with other groups (G, *P<0.05, NC versus ALS; ^$^*P*<0.05, SC versus ALS; ^#^*P*<0.05, NALS versus ALS). In contrast, IGF-1 expression was downregulated in the ALS group, although not significantly. *n*=5 in duplicates. Data were analysed using one-way ANOVA followed by Tukey's post hoc test.

The immunofluorescence analysis of BDNF showed minimal expression in the NC group (Fig. 5A). The expression was increased in the ALS group (Fig. 5D). BDNF expression was found to be uniformly distributed along the sarcolemmal region, and there was a punctate staining pattern in the sarcoplasm. The co-labelled BDNF and IGF-1 for the NC (Fig. 5C) and ALS (Fig. 5F) groups are provided, where the altered expression can be appreciated better. The quantification of BDNF expression confirmed the increased expression in the ALS group (Fig. 5G; *P<0.05, NC versus ALS, ^$^*P*<0.05, SC versus ALS, ^#^*P*<0.05, NALS versus ALS). Western blot analysis also showed a significant increase in the expression of BDNF (Fig. S1A,B). In contrast, immunoreactivity for IGF-1 in control muscles showed an intense sarcoplasmic and sarcolemmal distribution (Fig. 5B). However, IGF-1 expression showed a marginal downregulation in the ALS-CSF-injected group compared with the control groups (Fig. 5E,H). Further, we confirmed the trend of downregulation by immunoblotting for IGF-1 (Fig. S1A,C).

### Upregulated GDNF expression and downregulated VEGF expression

**Fig. 6.**
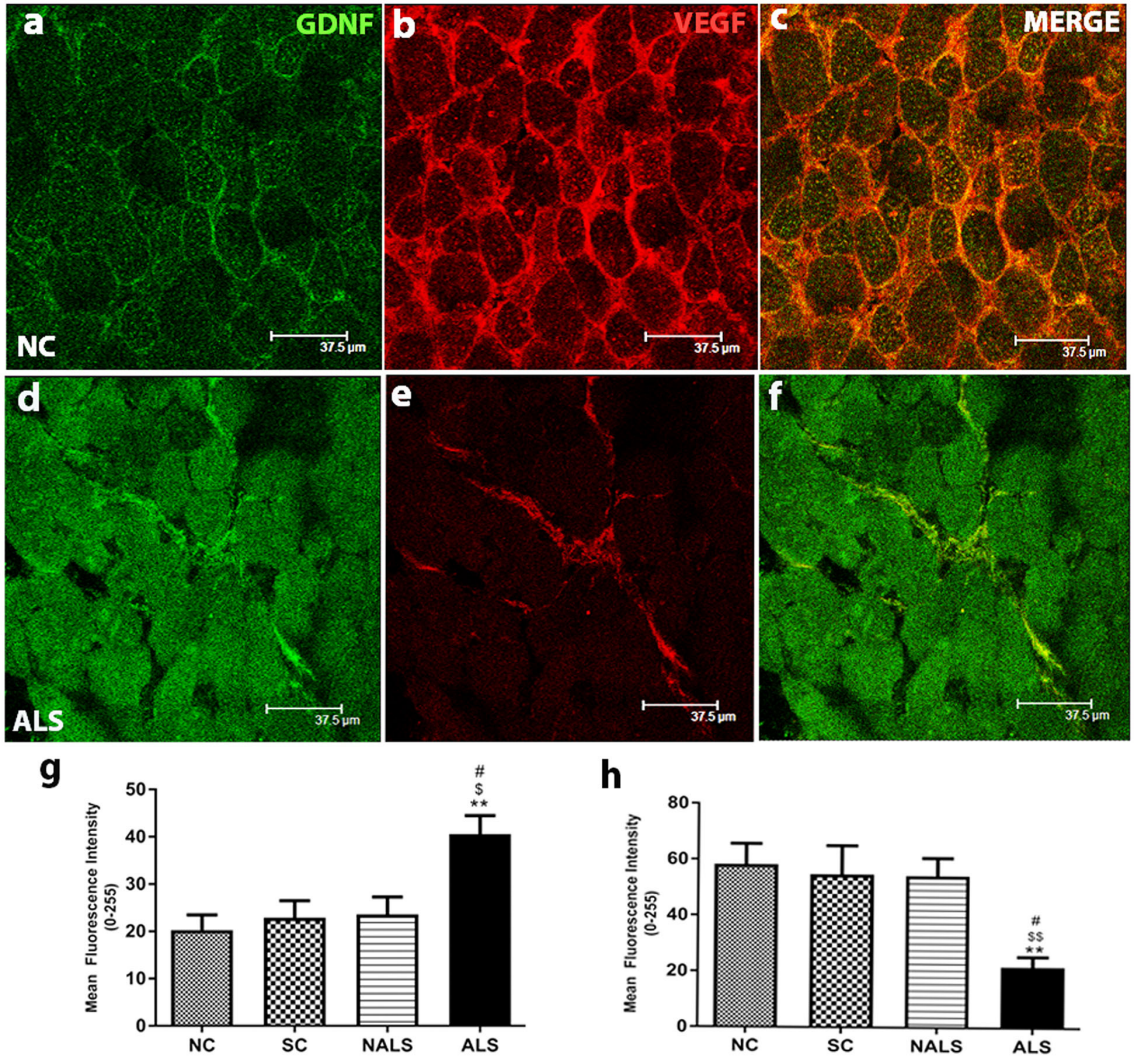
**Upregulated GDNF and downregulated VEGF expression.** (A-F) Representative photomicrographs of transverse muscle sections stained for GDNF (FITC) and VEGF (CY-3), showing altered expression. Increased and decreased expression of GDNF and VEGF, respectively, was seen in the muscle of ALS rats. Scale bars: 37.5 μm. (G,H) Quantification of immunofluorescence intensity supported the qualitative observations. GDNF showed a significant upregulation (G, **P<0.01, NC versus ALS; ^$^*P*<0.05, SC versus ALS; ^#^*P*<0.05, NALS versus ALS), whereas VEGF showed a significant downregulation (H, **P<0.01, NC versus ALS; ^$$^*P*<0.01, SC versus ALS; ^#^*P*<0.05, NALS versus ALS), in the ALS group compared with the other groups. *n*=5 in duplicates. Data were analysed using one-way ANOVA followed by Tukey's post hoc test.

GDNF was primarily localised to sarcolemma and a minimal punctuate staining was observed in the sarcoplasm (Fig. 6A). However, GDNF expression was significantly upregulated in the ALS group compared with the control groups, with uniform immunostaining even in the sarcoplasm (Fig. 6D). The co-labelled GDNF and VEGF for the NC (Fig. 6C) and ALS (Fig. 6F) groups are provided, where the altered expression can be appreciated better. The quantification of mean fluorescence intensity confirmed this finding (Fig. 6G; **P<0.01, NC versus ALS; ^$^*P*<0.05, SC versus ALS, ^#^*P*<0.05, NALS versus ALS). Western blot analysis of GDNF showed the trend for upregulation, although not significant (Fig. S1A,D). Immunofluorescence analysis of VEGF protein in control samples showed immunoreactivity in the sarcolemmal region and more prominently in the extracellular space (Fig. 6B). Further, the quantification showed a significantly decreased expression in the ALS group compared with the other groups (Fig. 6E,H; **P<0.01, NC versus ALS, ^$$^*P*<0.01, SC versus ALS, ^#^*P*<0.05, NALS versus ALS). Consistent with the immunohistochemical results, expression of VEGF was found to be significantly downregulated in the western blot analysis. (Fig. S1A,E).

## DISCUSSION

In the present study, we report neurogenic atrophy of muscle fibres, reduced structural complexity of NMJs along with denervation, altered levels of receptor-clustering proteins of NMJs, rapsyn and calpain, ultrastructural damage, increased oxidative stress and impaired expression of trophic factors in EDL muscle of ALS-CSF-injected rats. Taken together, these acute pathological changes suggest that skeletal muscle is significantly involved in the pathogenesis of SALS.

In mSOD1 mice, motor unit numbers are reduced, specifically in the fast-twitch muscle; however, in slow-twitch muscle, these changes are shown to develop only at the later stage of the disease (Hegedus et al., 2007, 2008; Kennel et al., 1996). These fast-twitch motor units innervate the type 2 muscle fibres, and hence in ALS, there is a selective vulnerability of these fibres (Atkin et al., 2005; Derave et al., 2003; Frey et al., 2000). Our finding of pathological changes in type 2 fibres in ALS-CSF-injected animals proves their vulnerability to ALS-CSF-induced toxicity. Further, grouping of type 2 fibres suggests the sprouting of motor axon terminals, a compensatory response to denervation.

The early-occurring symptoms in ALS provide evidence for distal axonopathy in this disease (Fischer et al., 2004; Frey et al., 2000; Narai et al., 2009; Rocha et al., 2013). In the present study, the structural complexity of NMJs was significantly compromised in ALS-CSF-treated animals. There was also increased denervation of the NMJs, suggesting the disruption of neuromuscular transmission. In addition, the significant decrease in rapsyn, a key molecule for clustering of AChRs, and a concomitant increase in calpain, a Ca^2+^-dependent protein, which acts against clustering, result in dispersed AChR clusters and fragmented NMJs (Chen et al., 2007). We believe that the trigger for NMJ damage in our model could be the denervation caused by the onset of degeneration in a small number of motor neurons, which is perhaps further aggravated by other pathological changes (discussed below).

The ultrastructural studies showed distinct changes, such as marked size variations in muscle fibres, fibre angulation, sarcolemmal folds and Z-line streaming, in the ALS group, confirming the muscle damage. Z-line streaming represents an altered expression of myofilaments, implying denervation (Massa et al., 1992). Interestingly, similar observations were earlier reported in ALS patients as well as in animal models (Iwasaki et al., 1991; Kristmundsdottir et al., 1990; Kumamoto et al., 1979; Visani et al., 2011).

Morphological abnormalities in mitochondria, such as giant mitochondria, paracrystalline inclusions, abnormal cristae and aggregation, have been reported previously (Chung and Suh, 2002; Dobrowolny et al., 2008). Our results are also in agreement with the above findings. Further, the SR regulates the calcium levels in the muscle in close association with the mitochondria (Rossi et al., 2009). Increased calcium signalling along with mitochondrial damage is also reported in the skeletal muscle of models of ALS (Kawamata and Manfredi, 2010; Zhou et al., 2010). Thus, calcium dysregulation plays a key role in ALS pathogenesis (reviewed in Grosskreutz et al., 2010). In the present study, the skeletal muscle of ALS animals exhibited dilated SR, damaged mitochondria and increased calpain expression, suggesting abnormal calcium homeostasis.

The ultrastructural finding of damaged mitochondria suggests an impaired redox status of the system. Oxidative stress leads to significant lipid peroxidation and we confirmed the same in the skeletal muscle of ALS-CSF-injected animals. Further, we observed downregulated activity of SOD, which might exacerbate the oxidative stress in the muscle. However, enhancement of the activity of thioredoxin reductase probably suggests a compensatory response. Similar upregulation of antioxidant enzyme activity, probably in response to an enhanced oxidative stress, is reported in the skeletal muscle of rodent models (Dobrowolny et al., 2008; Leclerc et al., 2001; Mahoney et al., 2006). The absence of an increase in SOD activity in our model contrasts with the increased SOD activity observed in a model of familial ALS (FALS) (Dal Canto and Gurney, 1995). This increase in SOD activity might be associated with the overexpression of 25 copies of the *SOD* gene in the FALS model, resulting in toxic ‘gain of function’. Antioxidant enzymes are required for maintaining the structural integrity of NMJs, and oxidative stress can impair neuromuscular transmission, as shown by G93A-SOD1 mice exhibiting a significant decrease in the release of neurotransmitters at NMJs (Naumenko et al., 2011; Sakellariou et al., 2014). Thus, the above findings confirm that oxidative stress is a major contributory factor to the NMJ degeneration seen in ALS (Pollari et al., 2014). Accordingly, in the current study, we propose that increased oxidative stress could be accelerating NMJ damage.

BDNF is differentially regulated in ALS as there are decreased levels of BDNF in the spinal cord and elevated levels in the skeletal muscle (Deepa et al., 2011; Küst et al., 2002; Nishio et al., 1998). The present study provides experimental evidence for elevated BDNF levels in the muscles of ALS-CSF-treated rats. This increase is either a compensatory response or a consequence of degeneration of motor neurons, leading to neurotrophin accumulation in the target skeletal muscle. Nevertheless, the increase in BDNF expression is likely to be transient, in view of the gradual decrease in BDNF as the disease progresses (Küst et al., 2002). The motor neurons can differentially regulate growth factor expression in skeletal muscle to promote regeneration of injured peripheral nerves (Funakoshi et al., 1995; Gómez-Pinilla et al., 2001). Thus, upregulated BDNF can be an initial compensatory mechanism provided by the skeletal muscle to rescue the degenerating motor neurons.

IGF-1 maintains the integrity of muscles and enhances satellite cell activity in mSOD1 mice (Dobrowolny et al., 2005). Decreased IGF-1 levels are seen in the spinal cord of ALS patients as well as in ALS-CSF-injected rats (Deepa et al., 2011; Wilczak et al., 2003). In the present study, IGF-1 expression was downregulated in the skeletal muscle of the ALS rats, similar to findings reported earlier in the skeletal muscle of ALS patients (Lunetta et al., 2012). Inflammatory response occurring in the skeletal muscle, such as increased expression of TNF-α, IL-6 and other cytokines, can inhibit IGF-1 expression (Frost et al., 2003; Street et al., 2006; Van Dyke et al., 2016; Wolf et al., 1996). Further, oxidative stress has the propensity to impair *IGF-1* mRNA expression in muscle culture (Sestili et al., 2009). Thus, reduced IGF-1 levels observed in the present study might be caused by oxidative stress in skeletal muscle. Considering the significant role of IGF-1 in neuronal survival, this reduction could affect the survival of motor neurons.

GDNF is a trophic factor mainly involved in NMJ formation (Wright and Snider, 1996). GDNF expression is increased in denervated skeletal muscle (Henderson et al., 1994; Lie and Weis, 1998; Zhao et al., 2004). Elevated *GDNF* mRNA expression is observed in the spinal cord (Yamamoto et al., 1996) as well as in skeletal muscle of ALS patients (Grundström et al., 1999; Lie and Weis, 1998; Yamamoto et al., 1999). It is a potential therapeutic agent, and adeno-associated virus-GDNF-treated ALS mice show a delayed disease onset and progression of motor dysfunction, along with prolonged life span (Wang et al., 2002). The significant increase in GDNF expression in the skeletal muscle of the ALS-CSF-injected rats is perhaps a transient compensatory mechanism to promote re-innervation of motor neurons. However, it could also be a generalised response to denervation injury (Lie and Weis, 1998; Zhao et al., 2004). Increased expression of c-Ret, a GDNF receptor, in motor neurons in ALS suggests that skeletal muscle probably attempts to compensate and prevent motor neuron degeneration by upregulating GDNF, but eventually it fails in its efforts (Mitsuma et al., 1999; Ryu et al., 2011). Further, the transient nature of GDNF upregulation is shown in the autopsied skeletal muscles of ALS patients, where skeletal muscles with severe fibre depletion show reduced levels of *GDNF* mRNA (Yamamoto et al., 1999, 1996).

Altered VEGF levels in the serum, CSF and anterior horn cells of ALS patients, and also in the spinal cord of mSOD1 mice, have been reported (Brockington et al., 2006; Gao et al., 2014; Gupta et al., 2011; Lunn et al., 2009). Moreover, therapeutic potential of VEGF is shown using rodent models by retrograde delivery and by exogenous supplementation to NSC-34 motor neurons (Azzouz et al., 2004; Krakora et al., 2013; Kulshreshtha et al., 2011; Vijayalakshmi et al., 2015). Further, VEGF offers neuroprotection by enhancing axonal outgrowth (Carmeliet and Ruiz de Almodovar, 2013). Hence, decreased VEGF expression might lead to significant muscle damage, thus accelerating motor neuronal loss.

**Fig. 7.**
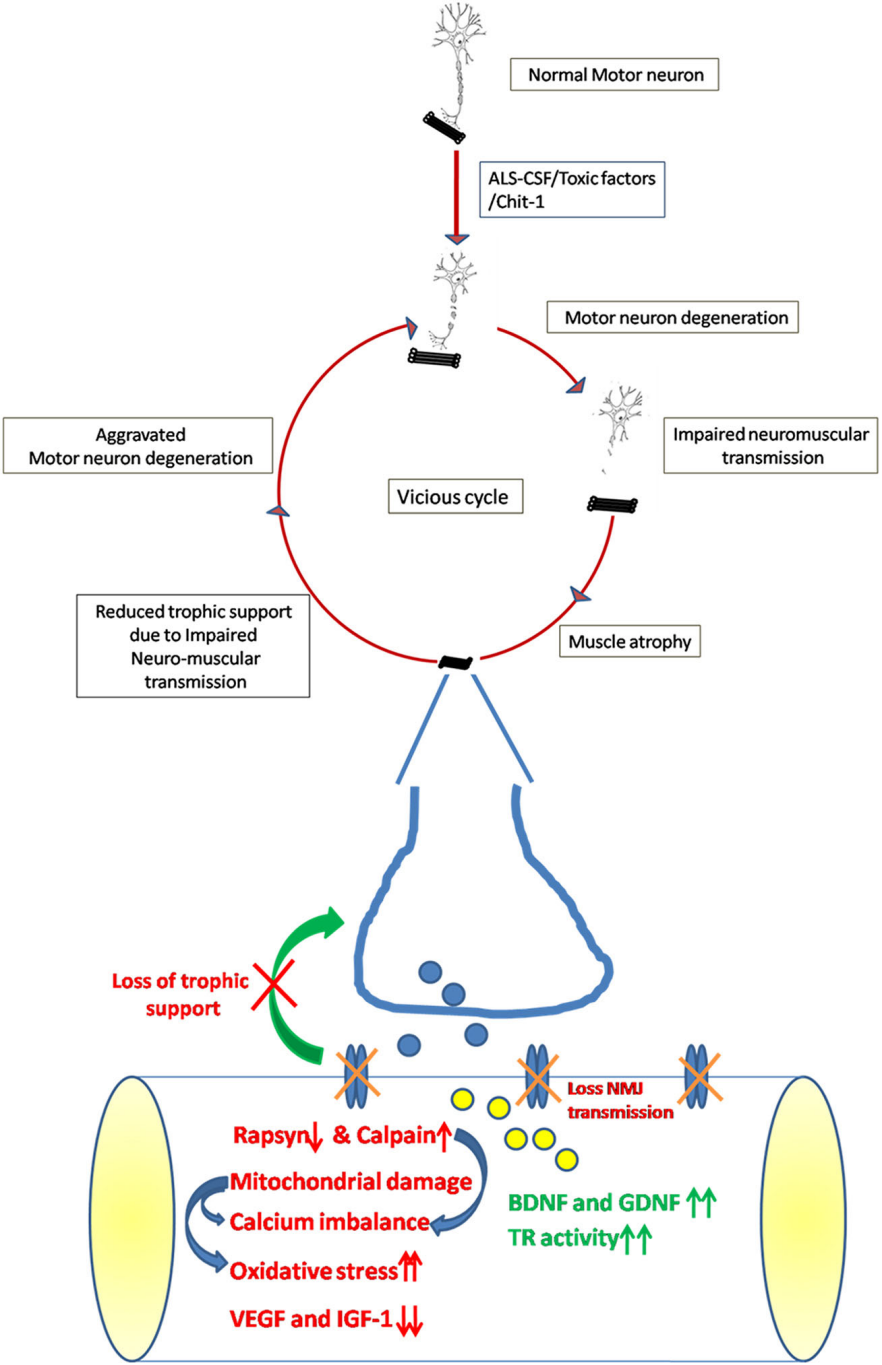
**Schematic representation of the contribution of skeletal muscle in SALS pathogenesis.** The early insult to motor neurons caused by the intrathecal injection of ALS-CSF results in impaired neuromuscular transmission, causing the skeletal muscle to undergo alterations, such as muscle atrophy, disintegration and disruption of NMJ. The skeletal muscle also showed increased oxidative stress as a result of mitochondrial damage and impaired calcium homeostasis, as suggested by dilated sarcoplasmic reticulum and increased expression of calpain. Expression of IGF-1 and VEGF was also reduced, thus depleting the major trophic support. Nevertheless, the muscle initially attempts to offer neuroprotection by upregulating the levels of BDNF and GDNF but, owing to damaged NMJ, motor neurons are unable to capitalise on it. Deprivation of trophic support results in the residual surviving motor neurons, which escaped the initial insult, to undergo degeneration, thus resulting in the relentless progress of ALS.

The present study attempts to give a comprehensive account of changes seen in the skeletal muscle of neonatal rats injected with CSF from SALS patients (Fig. 7). These include loss of NMJ and atrophy of muscle. The skeletal muscle affected by exposure to ALS-CSF attempts to confer protection to degenerating motor neurons by upregulating BDNF and GDNF, but this is countered by a loss of neuromuscular synapses and decreased levels of IGF-1 and VEGF. All these changes might aggravate the degeneration of surviving motor neurons, thus initiating a vicious cycle, leading to rapid progression of this disease.

## MATERIALS AND METHODS

### CSF sample collection

CSF samples from clinically confirmed ALS patients [El Escorial criteria (Brooks et al., 2000)], were collected through lumbar puncture by a neurologist. The Human Ethics Committee of the National Institute of Mental Health and Neurosciences, Bengaluru, approved the use of human CSF samples for the study (Item no. III, SI no. 3.01, Basic Sciences), and consent was obtained from all participants prior to CSF collection. Age- and gender-matched patients suffering from non-neurodegenerative, noninfectious neurological diseases, such as benign intracranial hypertension and transverse myelitis, were included as non-ALS controls. The CSF samples were snap frozen in liquid nitrogen and stored at −80°C until use.

### Intrathecal injection procedure

Neonatal Wistar rats required for the study were procured from the Central Animal Research Facility of the National Institute of Mental Health And Neurosciences, Bengaluru, subsequent to approval by the Institutional Animal Ethics Committee (IAEC) [AEC/52/324/NP, AEC/56/324(B)/NP and AEC/60/324(C)/NP]. The animals were handled in accordance with National Institutes of Health (NIH) guidelines. Intrathecal injections were carried out as described previously (Rao et al., 1995). Briefly, 3-day-old Wistar rat pups were deeply anesthetised with halothane and a dorsal midline skin incision (1 mm) was made about 1 cm rostral to the base of the tail. Using a microinjector, 5 µl of CSF was intrathecally injected into the subarachnoid space at a rate of 1 µl/2.5 min. The incision was sutured, cleaned and sprayed with Healex (Rallis, India), an anti-inflammatory liquid. The injections were carried out on postnatal days 3, 9 and 14. The animals were sacrificed on postnatal day 16, and the whole extensor digitorum muscle was dissected out carefully and snap frozen in isopentane pre-cooled in liquid nitrogen, for enzyme histochemistry and immunohistochemistry, and fixed in 3% glutaraldehyde for electron microscopy.

The animals were grouped as follows: (1) normal control (NC), animals that were not subjected to the injection procedure; (2) sham control (SC), animals subjected to the sham injection procedure; (3) non-ALS-CSF (NALS), animals injected with non-ALS-CSF samples; (4) ALS-CSF (ALS), animals injected with ALS-CSF samples.

### ATPase staining

The EDL muscle was flash frozen in isopentane, pre-cooled in liquid nitrogen, and serial cryosections (8 µM) were collected on glass slides. The cryosections were incubated with pre-incubating solution (44 mg CaCl_2_, 41 mg sodium barbiturate in 10 ml distilled water, pH 9.4) for 20 min at 37°C. This was followed by exposure to the incubating solution (19.98 mg CaCl_2_, 20.6 mg sodium barbiturate, 2 mg ATP salt in 10 ml distilled water, pH 9.6) for 45 min. The slides were then washed twice with 1% CaCl_2_, followed by three washes in 2% CoCl_2_ and two to three washes in distilled water. Later, the slides were developed in 1% yellow ammonium sulphide solution (freshly prepared), washed in double-distilled water, air dried and mounted in glycerine jelly.

### Electron microscopy

The whole EDL muscle was fixed in 3% buffered glutaraldehyde and post-fixed with 1% osmium tetroxide for 2 h at 4°C. The tissues were then dehydrated through a graded series of ethanol washes, cleared in propylene oxide, embedded in resin and left undisturbed at 60°C for 2 days to allow polymerisation. Ultrathin sections, contrasted with uranyl acetate and lead citrate, were viewed using a transmission electron microscope (FEI, TECNAI G^2^ Spirit BioTWIN, The Netherlands).

### Immunostaining of NMJ

Serial longitudinal cryosections of 40 µM thickness were collected on glass slides and fixed in 4% paraformaldehyde (PFA) for 10 min. The sections were incubated in 1.5% bovine serum albumin (BSA) followed by α-bungarotoxin (1:200, Invitrogen, USA) for 3 h at room temperature, washed in 0.1 M phosphate buffered saline (PBS), pH 7.4 and mounted. The sections were co-labelled with an antibody against 200 kD neurofilaments (1:1000, Abcam, USA) followed by a secondary antibody [anti-rabbit cyanine 3 (CY3), 1:1000] for easy detection of innervation-, denervation- and re-innervation-induced changes in NMJs.

### Biochemical analysis of oxidative stress and antioxidant enzyme activity

#### Preparation of whole muscle protein extracts

Frozen muscle tissue (50 mg) was thawed and minced in 10 volumes of 1× PBS containing protease inhibitors and homogenised. The samples were sonicated on ice in six cycles of 5 s each. The extract was centrifuged (14,000 ***g***, 10 min) and the supernatant was subjected to protein estimation by the Bradford method.

#### Bradford method

Briefly, 10 μl of the suitably diluted protein sample was pipetted into a 96-well ELISA plate to which 200 µl 1× Bradford reagent [100 mg Coomassie Brilliant Blue G-250, 50 ml ethanol (95%) and 100 ml orthophosphoric acid (85%) in 200 ml distilled water] was added, thoroughly mixed and incubated for 10 min. The absorbance was read at 595 nm in an ELISA plate reader (TECAN, Austria). The concentration of protein in the unknown sample was calculated as compared to BSA standards (50-500 μg/ml). All estimations were performed in triplicate.

#### Estimation of lipid peroxidation

The extracted protein samples (2000 μg) were added to TBA/TCA reagent (7.5 mg thiobarbituric acid, 300 μl 100% trichloroacetic acid, 256 μl 1 N HCl). The mixture was then heated for 20 min in boiling water, after which the samples were subjected to centrifugation at 955.89 ***g*** for 10 min. The supernatant was collected and absorbance measured at 532 nm. MDA concentration was calculated using the molar extinction co-efficient (MEC) (241 mol/cm) and normalised per mg protein.

#### SOD assay

For SOD assay, 10 μl of the supernatant (100 μg protein) was mixed with 30 mM Tris-HCl buffer (pH 9.1), 0.5 mM ethylenediaminetetraacetic acid (EDTA), 50 mM tetramethylethylenediamine (TEMED) and 0.05 mM quercetin. The rate of quercetin oxidation was monitored at 406 nm for 10 min (1 U SOD activity=amount of enzyme/mg protein that inhibits quercetin oxidation by 50%).

#### Thioredoxin reductase assay

The reaction mixture contained 0.2 M phosphate buffer, pH 7.4, 1 mM EDTA, 0.4 mM NADPH and sample (15 μl). The assay reaction was initiated by the addition of 10 μl of 2 mM 5,5′-dithio-bis-(2-nitrobenzoic acid) (DTNB) and 5-thio-2-nitrobenzoic acid formed was measured at 412 nm. The activity was expressed as nM of DTNB reduced/min/mg protein.

#### Glutathione reductase assay

For glutathione reductase assay, 10 μl of the supernatant (100 μg protein) was mixed with Tris-HCl buffer (0.1 M, pH 8.8), 0.1 mM EDTA and NADPH (0.2 mM). The reaction was initiated by adding 12.5 μl of G-S-S-G (oxidised glutathione) and the decrease in absorbance at 340 nm was monitored for 5 min. The enzyme activity was expressed as nM of NADPH oxidised/min/mg protein (MEC=6.220 M^−1^ cm^−1^).

### Immunoblotting

Total muscle protein was extracted from the muscle tissue (10 mg) with a solution containing 75 mM Tris-HCl, pH 6.8, 15% SDS, 20% glycerol, 5% dithiothreitol and 0.001% Bromophenol Blue, followed by sonication for 30 s, boiling at 95°C for 5 min and centrifugation at 15,000 ***g*** for 5 min. The supernatant was then used for immunoblotting of rapsyn [mouse anti-rapsyn (1:500), Abcam, ab11423], calpain [rabbit anti-calpain II, large subunit (1:500), Merck Millipore, USA, AB81023], BDNF [rabbit anti-BDNF (1:500), Santa Cruz Biotechnology, USA, sc-546], IGF-1 [goat anti-IGF-1 (1:500), Santa Cruz Biotechnology, sc-7144], GDNF [mouse anti-GDNF (1:200), Santa Cruz Biotechnology, sc-13147] and VEGF [rabbit anti-VEGF (1:500), Santa Cruz Biotechnology, sc-507] proteins. For the loading control, protein extracts were run on 6% SDS-PAGE followed by Coomassie Brilliant Blue staining.

### Immunohistochemical labelling of trophic factors

Cryosections (8 µM) of EDL muscles were equilibrated with 0.1 M PBS (pH 7.4) at room temperature and post-fixed with 2% PFA for 15 min, followed by blocking in 3% BSA for 2 h. Subsequently, the sections were incubated with the first primary antibody [rabbit anti-BDNF (1:500), Santa Cruz Biotechnology, sc-546 or rabbit anti-GDNF (1:500), Santa Cruz Biotechnology, sc-328] for 48 h. The sections were washed in PBS and incubated with fluorochrome-conjugated secondary antibody [anti-rabbit fluorescein isothiocyanate (FITC) (1:1000) or anti-rabbit Cy3 (1:1000)] for 4 h at room temperature. The sections were re-equilibrated with PBS followed by blocking in 3% BSA for 2 h at room temperature. Further, the sections were incubated with the second primary antibody [goat anti-IGF-1 (1:500), Santa Cruz Biotechnology, sc-7144 or rabbit anti-VEGF (1:500), Santa Cruz Biotechnology, sc-507] for 48 h, washed in PBS and incubated with fluorochrome-conjugated secondary antibody [anti-goat Cy3 (1:1000) or anti-rabbit FITC (1:1000)] at room temperature for 4 h. Stained sections were mounted onto cover slips in 65% glycerol and processed for image capturing with a confocal laser microscope (DMIRE-TCS, Leica, Germany) (Vijayalakshmi et al., 2015).

### Imaging and quantification of immunofluorescence

Total NMJ area and AChR protein expression were measured using in-built Leica software of the confocal microscope by demarcating the edges of the α-bungarotoxin-labelled NMJs using the poly-line profile of the program. The total area within the marked boundary was measured to obtain the corresponding numerical values commensurate to the staining intensity of α-bungarotoxin-labelled NMJs. Similarly, for the quantification of trophic factors, each myofibre in the transverse section was demarcated and the image was analysed. The quantification was performed on a scale of 0-255, where 0 depicts absence of staining and 255 represents the most intense staining. The quantitative analysis was carried out on 10 sections of the EDL muscle per animal, and data from five pairs of animals were considered for each group.

### Statistical analysis

Each experimental group consisted of 10 rats. Five different CSF samples were used in duplicate for all experiments. Statistical analysis was carried out using one-way analysis of variance (Naumenko et al., 2011) followed by Tukey's post hoc test. *P*<0.05 was considered significant. Data are expressed as mean s.e.m.

## Supplementary Material

Supplementary information

First Person interview
