# Supplementary material for: Sporadic amyotrophic lateral sclerosis (SALS) – skeletal muscle response to cerebrospinal fluid from SALS patients in a rat model
Source: Dis Model Mech. 2018 Apr 16;11(4):dmm031997. doi: 10.1242/dmm.031997 (PMC5963857; doi:10.1242/dmm.031997)
Supplement: Supplementary information [file dmm-11-031997-s1.pdf]

© 2018. Published by The Company of Biologists Ltd | Disease Models & Mechanisms (2018): doi:10.1242/dmm.031997: Supplementary information

FIRST PERSON

First person – Shruthi Shanmukha

First Person is a series of interviews with the first authors of a selection of papers published in Disease Models & Mechanisms, helping early-career researchers promote themselves alongside their papers. Shruthi Shanmukha is first author on 'Sporadic amyotrophic lateral sclerosis (SALS) – skeletal muscle response to cerebrospinal fluid from SALS patients in a rat model', published in DMM. Shruthi is a PhD student in the lab of Dr Trichur R. Raju at National Institute of Mental Health And Neurosciences, Bangalore, India, investigating the molecular mechanism of neurodegenerative disease, signalling pathways in the nervous system and their contribution to disease, trophic factors in signalling, and mitochondrial physiology.

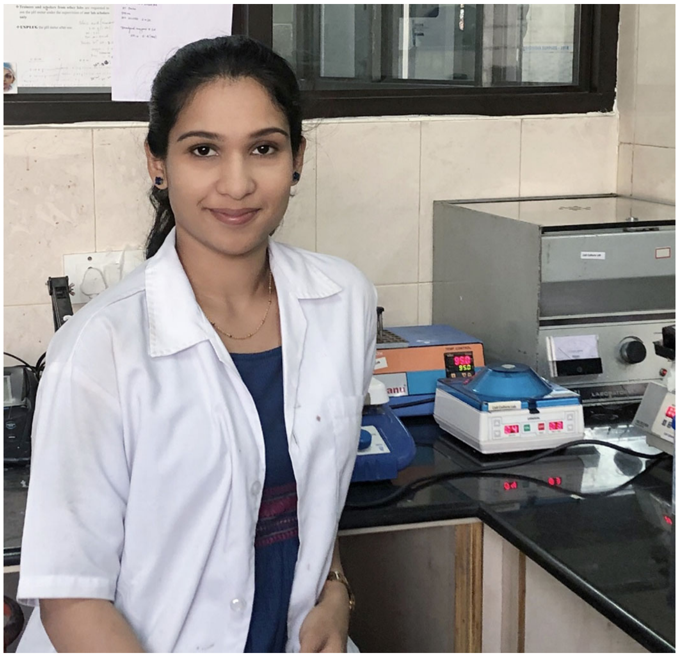

Shruthi Shanmukha

How would you explain the main findings of your paper to non-scientific family and friends?

Amyotrophic lateral sclerosis (ALS) is a neurological disease which is characterized by the dysfunction and/or degeneration of neurons that control muscle movement. This causes muscle weakness, paralysis and eventually death within 3-5 years of onset, mainly due to respiratory failure. In 95% of cases, ALS is sporadic, where there is no known cause, and the remaining cases are inherited or familial. However, the majority of research on ALS is being performed on a genetic model that represents the familial form of the disease. We have modelled the sporadic form of the disease by injecting cerebrospinal fluid (CSF) from ALS patients into young rats. We then extensively studied the skeletal muscle of these rats, and our study reveals that various pathological changes occurring in skeletal muscle can contribute significantly to disease progression.

What are the potential implications of these results for your field of research?

Research in the ALS field has always been motor neuron-centric. However, the non-cell-autonomous nature of the disease is widely accepted. Various cells such as astrocytes, microglia and skeletal muscle have a major role to play in disease progression. It is evident from our study that the various pathological changes to skeletal muscle significantly aggravate this disease progression. Aside from unraveling the pathomechanisms, our study further recommends skeletal muscle-based therapies as a potential curative.

What are the main advantages and drawbacks of the model system you have used as it relates to the disease you are investigating?

The main advantage of our model is that it represents the sporadic model of ALS that constitutes about 90% of cases. There is no major drawback, but the degree of neurotoxicity in the model varies from sample to sample of CSF. Conversely, this variation can be considered to be an advantage as this makes our model patient-specific.

What has surprised you the most while conducting your research?

Our initial hypothesis was that there is decreased growth factor expression during the course of the disease. However, it turned out that not all the trophic factors were downregulated; in fact, BDNF and GDNF were upregulated compared with the normal control group, suggesting an active compensatory role played by skeletal muscle under disease conditions.

Describe what you think is the most significant challenge impacting your research at this time and how will this be addressed over the next 10 years?

Currently, there is no cure for ALS and until recently the only FDA-approved drug was riluzole, which increases lifespan by approximately two to three months. Very recently one more drug, edaravone, was approved by the FDA, but its long-term safety and efficacy are not yet clear. Understanding the pathomechanisms of ALS is key to finding effective therapies. I personally feel that this problem will be addressed over the next 10 years only through better collaboration between clinicians and research scientists, and by more translational research.

What changes do you think could improve the professional lives of early-career scientists?

Early-career scientists needs to have good fellowship support to be motivated in research. In addition, travel grants are essential so that they can interact with different researchers and gain information around the globe. Further, I feel that short-term fellowship programmes to visit different labs and to learn new skills would really enhance researchers' technical knowledge, giving new perspective and resulting in better research.

Shruthi Shanmukha's contact details: Department of Neurophysiology, National Institute of Mental Health And Neurosciences, Bangalore 560 029, India.
E-mail: shruthishanmuk@gmail.com

“I feel that short-term fellowship programmes to visit different labs and to learn new skills would really enhance researchers’ technical knowledge”

What’s next for you?

My research experience so far has been interesting and I would like to study the cellular and molecular mechanisms underlying neurological diseases further. Currently, my goal is to find an interesting postdoctoral position in neuroscience that offers an interdisciplinary research environment.

Reference

Shanmukha, S., Narayanappa, G., Nalini, A., Alladi, P. A and Raju, T. R. (2018). Sporadic amyotrophic lateral sclerosis (SALS) – skeletal muscle response to cerebrospinal fluid from SALS patients in a rat model. *Dis. Model. Mech.* **11**: dmm031997, doi:10.1242/dmm.031997.
